# Macrophages and Galectin 3 Control Bacterial Burden in Acute and Subacute Murine Leptospirosis That Determines Chronic Kidney Fibrosis

**DOI:** 10.3389/fcimb.2018.00384

**Published:** 2018-10-30

**Authors:** María F. Ferrer, Emilia Scharrig, Nancy Charo, Ana L. Rípodas, Ricardo Drut, Eugenio A. Carrera Silva, Ariel Nagel, Jarlath E. Nally, Daniela P. Montes de Oca, Mirta Schattner, Ricardo M. Gómez

**Affiliations:** ^1^Laboratory of Animal Viruses, Institute of Biotechnology and Molecular Biology, UNLP-CONICET, La Plata, Argentina; ^2^Laboratory of Experimental Thrombosis, Institute of Experimental Medicine, National Academy of Medicine-CONICET, Buenos Aires, Argentina; ^3^BioLab, La Plata, Argentina; ^4^Division of Pathology, Children Hospital “Superiora Sor María Ludovica”, La Plata, Argentina; ^5^Biotechnology Institute, National Institute of Agricultural Technology (INTA-CONICET), Buenos Aires, Argentina; ^6^Infectious Bacterial Diseases Research Unit, National Animal Disease Center, Agricultural Research Service, United States Department of Agriculture, Pullman, WA, United States; ^7^Ecology, Genetics and Evolution Department, Exact and Natural Sciences Faculty, and Ecology, Genetics and Evolution Institute of Buenos Aires, UBA-CONICET, Buenos Aires, Argentina

**Keywords:** macrophages, galectin 3, fibrosis, *Leptospira*, pathogenesis

## Abstract

Previous studies have suggested that macrophages may contribute to acute *Leptospira* dissemination, as well as having a major role in kidney fibrosis. Our aim was to characterize the role of macrophages and galectin 3 (Gal-3) on the survival, clinical course, bacterial burden, interstitial nephritis, and chronic kidney fibrosis in *Leptospira interrogans* serovar Copenhageni (LIC)-induced experimental murine leptospirosis. C57BL/6J mice depleted of macrophages by liposome-encapsulated clodronate treatment and infected with LIC presented a higher bacterial burden, had reduced subacute nephritis and enhanced chronic kidney fibrosis relative to untreated, infected mice. Moreover, LIC infection in mice whose Gal-3 was disrupted (*Lgals3*^−^^/–^) had a higher bacterial burden and enhanced subacute nephritis and chronic kidney fibrosis when compared to C57BL/6J wild-type mice. Chronic fibrosis did not correlate with higher transcription levels of TGF-β1 or IL-13 in the kidneys. Kidney fibrosis was found in chronically infected rats as well as in wild infected rats. On the other hand, human fibroblast cultures exhibited enhanced differentiation to myofibroblasts after treatment with LIC. Our results demonstrate that macrophages and Gal-3 play a critical role in controlling the LIC burden but has a minor role in subsequent fibrosis. Instead, kidney fibrosis was better correlated with bacterial burden. Taken together, our results do not support a role for macrophages to disseminate leptospires during acute infection, nor in chronic kidney fibrosis.

## Introduction

Leptospirosis is a global zoonosis caused by pathogenic spirochetes of the genus *Leptospira* that frequently occurs in tropical areas (Bharti et al., 2003). Recently, it has been estimated that worldwide there are more than 1,000,000 cases and nearly 60,000 deaths due to leptospirosis each year (Costa et al., 2015). The transmission is frequently related to contact with urine-contaminated soil and water or with infected animal tissues (Faine, 1988; Lecour et al., 1989). In addition, leptospirosis is currently recognized as a neglected disease associated with poverty and slum settlements where sanitation is lacking (Hartskeerl et al., 2011; Sarkar et al., 2012; Felzemburgh et al., 2014).

The pathogenesis of leptospirosis is not completely known (Haake and Levett, 2014). Pathogenic *Leptospira* spp. disseminate during the acute leptospiremic phase of infection that may affect kidney physiology (Wunder et al., 2016). The subacute leptospiruric phase begins 5–12 days later with an increase in specific antibodies that clear the bacteria from blood and other organs. However, bacteria are not cleared from the kidneys, where they can chronically persist in the proximal tubules which results in shedding of leptospires into the urine for months producing a chronic inflammation and the host becomes a carrier that contaminates the environment with their urine (Haake and Levett, 2014).

In the past, the role of monocytes and derived macrophages was studied for many diseases after their depletion using silica particles (Allison et al., 1966). This procedure was later associated with increased macrophage activation (Van Rooijen and Sanders, 1997), which led to uncertainty in the role of macrophages in many of those studies. More recently, the role of monocytes and macrophages in different pathologies has been clarified by specific depletion of these cells using liposome encapsulated clodronate (LipClod) (Van Rooijen and Hendrikx, 2010). However, their role in leptospirosis is still unclear, even with a study showing that the silica particles treatment did not affect the outcome of the infection (Tu et al., 1982). Another study showed that *Leptospira* may persist and even replicate in macrophages and proposed that macrophages might be carriers of leptospires and aid in their spread to target organs (Toma et al., 2011).

Galectin 3 (Gal-3) belong to the family of β galactoside binding animal lectins, and it is highly expressed and secreted by monocytes and activated macrophages functioning as a regulator of acute and chronic inflammation (Liu and Rabinovich, 2010). In addition, Gal-3 secretion by macrophages is a major driver of fibroblast activation and subsequent myofibroblast accumulation during the progression of several organs to fibrosis (Henderson et al., 2006, 2008; Jaquenod De Giusti et al., 2015), plays a critical and complex context-dependent role in the kidneys (Chen and Kuo, 2016) and have an enhanced expression in chronic murine leptospirosis (Ferrer et al., 2014).

In the present study, we aimed to clarify the respective roles of macrophages and Gal-3 in controlling the bacterial burden, interstitial nephritis and renal fibrosis by performing a comparative study of *Leptospira interrogans* infection in normal, macrophage-depleted or Gal-3 depleted mice.

## Methods

### Bacteria

The virulent *L. interrogans* serovar Copenhageni (LIC) strain Fiocruz L1–130 has been described previously (Ko et al., 1999). Bacteria were cultured at 30°C under aerobic conditions in liquid Ellinghausen-McCullough-Johnson-Harris (EMJH) media (Difco, USA) supplemented with 10% rabbit serum (vol/vol), 0.015% L-asparagine (wt/vol), 0.001% sodium pyruvate (wt/vol), 0.001% calcium chloride (wt/vol), 0.001% magnesium chloride (wt/vol), 0.03% peptone (wt/vol), and 0.02% meat extract (wt/vol) (Pretre et al., 2013). The bacteria were quantified by direct counting in a Petroff-Hausser chamber (Hausser Scientific, UK) using dark field microscopy (Scharrig et al., 2015). Virulence of LIC was maintained by iterative passages in Golden Syrian hamsters and to reduce its pathogenic variability, aliquots were freezed at −80°C and when used, thawed was performed in 2 ml of medium that were cultured 7 days before inoculation.

### Animals and experimental design of animal infections

Ethics Statement: all animal experiments were conducted in compliance with the Argentine animal protection Law “Ley 14346—Malos tratos y actos de crueldad a los animales.” The Institutional Animal Care and Use Committee of the Facultad de Ciencias Exactas, Universidad Nacional de La Plata dependence has reviewed the protocol and procedure for the care and use of laboratory animals entitled “Chronic experimental infection of C57BL/6J mice with *L. interrogans*.” This Committee has found that the procedures are in agreement with local guidelines for vertebrate animal welfare as well as with US Public Health Service and/or European Union policy (National Research Council, National Academy Press, Washington DC, 2010, and/or European Union Directive for Animal Experiments 2010/53/EU). This project was approved under the Protocol Number 016-06-15. All infectious experiments have been conducted following IBBM institutional safety procedures and were performed under biosecurity level 2, as it is required for *L. interrogans*. All animals received water and food *ad libitum*. All efforts were made to minimize suffering.

Weanling (3–4 weeks old) C57BL/6J inbred male mice used for the macrophage depletion experiments were supplied by the Faculty of Veterinary, UNLP, Argentina. Gal-3 deficient mice (*Lgals3*^−/−^)(B6.Cg-*Lgals3*^*tm*1*Poi*/*J*^) and their control littermates with a C57BL/6J genetic background were originally obtained from Jackson Laboratory (stock 6338) and were kindly supplied by Dr. O. Campetella of the Instituto de Investigaciones Biotecnológicas (IIB), Universidad Nacional de San Martín (Buenos Aires). Mice were injected intraperitoneally (IP) with 0.2 mL of phosphate buffered saline (PBS, Mock) or 0.2 mL of PBS containing 10^7^ LIC. For macrophage depletion, mice were injected IP with 10 μL/g of animal weight of LipClod (clodronateliposomes.org) 24 h before infection and then every 5 days (Mock+LipClod and LIC+LipClod groups). Macrophage depletion was confirmed by peripheral blood smears and by immunocytochemical detection of the F4/80 antigen in tissues (Jaquenod De Giusti et al., 2015). Mice were monitored daily and euthanised by CO_2_ overdose at 14 and 45 dpi (6–8 mice were used for each time point and two independent experiments were performed) for collection of their blood and kidneys. Routinely, the kidneys were sliced in half along their long axis, and one half was frozen at −80°C for further studies while the other was fixed in 4% paraformaldehyde.

All experimentation in rats was conducted in accordance with protocols as reviewed and approved by the Animal Care & Use Committee at the National Animal Disease Center, and as approved by USDA Institutional guidelines. Female Fisher 344 inbred rats (Strain F344/NHsd, Envigo) of approximately 4 weeks of age were experimentally infected with 1 × 10^7^ low-passage *L. interrogans* serovar Copenhageni as described (Nally et al., 2018).

### Live-trapping of wild rats

Live-trapping of wild rats was performed in nine livestock farms in central Buenos Aires province during August 2016. Cage traps (15 × 16 × 31 cm) were placed in different environments of the farms for three consecutive nights. Traps were checked for captures every morning and captured animals were humanely sacrificed to collect tissue samples after a deep anesthesia with an intramuscular injection of ketamine-acepromazine. Trapping, handling, and euthanasia were done following the procedures and protocols approved by the Argentine Law for Animal Care 14346, the Argentinean Society for Mammalian Studies (www.sarem.org.ar), the American Society of Mammalogists (Sikes et al., 2016).

### Histopathology

Samples were processed for routine histology and kidney injury was scored by a professional pathologist as previously described (Ferrer et al., 2014). Briefly, nephritis was graded blindly by a pathologist on a scale of 0–4 in a whole longitudinal section of the organ: a score of 0 corresponded to the absence of inflammatory infiltrates or necrosis, 1 denoted minimal inflammation (1 to 5 foci), 2 indicated mild inflammation (<25% of the section was affected, or 6–10 foci), 3 suggested moderate inflammation (25–50% of the section was affected, or 11–20 foci), and 4 corresponded to severe inflammation (more than 50% of the section had inflammatory infiltrates, or more than 20 foci). Picrosirius red staining and digital image analysis were used to quantify the amount of red-stained collagen fibers as previously described (Ferrer et al., 2014) using a Nikon E200 microscope with a Tucsen TCC 5.0 digital camera and the software provided by the manufacturer. Briefly, the full kidney was photographed (usually 70–80 pictures), and 5 pictures of each sample with the larger vessels were discarded. Background levels were set for all the experiment pictures. Measurement of collagen deposits was performed using the green channel. Percentage of collagen staining was obtained for each picture.

### Immunohistochemistry

Immunohistochemistry was performed as previously described (Ferrer et al., 2014). Briefly, rehydrated Pro-Bond Plus tissue sections were heated three times for 5 min in a 10 mmol/L citrate buffer in a microwave oven. The sections were then cooled, immersed in 3% H_2_O_2_ for 15 min, and incubated in 5% normal goat serum in PBS for 20 min at room temperature. The samples were incubated with a primary murine polyclonal anti-LipL32 antibody, generously provided by Dr. Nascimento of Butantan Institute, the mouse monoclonal anti-macrophage F4/80 antibody (Novus Biologicals, USA) or the anti-α-smooth muscle actin protein (Clone 1A4, Dako, USA) for 1 h at room temperature. Then, samples were incubated with a secondary anti-mouse antibody conjugated to a peroxide-labeled dextran polymer (Dako EnVision, CA, USA). The hydrogen peroxidase substrate diaminobenzidine was added for 10–20 min to reach the appropriate intensity and then the slides were rinsed with distilled water. Immunostained sections were counterstained with hematoxylin for 1 min, washed with tap water, rinsed with distilled water, and dehydrated in increasing concentrations of ethanol followed by xylene (each treatment was 5 min). Slides were then observed under a Nikon E200 photomicroscope.

### DNA isolation

DNA was isolated as previously described (Ferrer et al., 2014). Briefly, kidney samples were subjected to mechanical homogenisation in 500 μL of lysis buffer (50 mM Tris-HCl pH 8.0, 1 μM EDTA, 1% Triton X-100, 0.5% Tween-20, 1% SDS) containing proteinase K (2 μg/mL) and incubated for 2 h at 56°C. The reaction was mixed with one volume of phenol (pH 8.0) and centrifuged at 12,000 × g for 15 min at 4°C. The aqueous phase was extracted once more with phenol and then four times with 0.5 volumes of chloroform/isoamyl alcohol (24:1), with centrifugation at 12,000 × g for 10 min at 4°C between each extraction. DNA was precipitated by the addition of 100 μL 3 M sodium acetate pH 5.2 and 100% ethanol up to a volume of 1 mL and stored overnight at −20°C. DNA was pelleted by centrifugation at 12,000 × g for 20 min at 4°C. The DNA pellet was washed twice with 70% ethanol, resuspended in TE buffer (10 mM Tris-HCl pH 7.5, 1 mM EDTA) and incubated at 55–60°C for 15–20 min. DNA was stored at −20°C until use.

### Real time-PCR

Real time-PCR (qPCR) studies were performed with a Line-Gene K instrument and software (Bioer) as described (Ferrer et al., 2014). The 5X HOT FIRE Pol Eva Green qPCR Mix Plus was used for all reactions according to the manufacturer's instructions. Initial denaturation was carried out at 94°C for 10 min, followed by 65 cycles of 20 s at 94°C, 15 s at the respective annealing temperature and 15 s at 72°C for each round of extension, followed by a final extension at 72°C for 2 min. A melting curve analysis was performed immediately after amplification, ranging from 70 to 89°C with a linear temperature transition rate of 0.3°C/s and continuous fluorescence acquisition. The size of PCR products was confirmed by agarose gel electrophoresis. To calculate the bacterial burden, the 16S DNA bacterial gene was amplified, quantified and results were expressed as the number of *Leptospira* equivalent genomes per mg of kidney tissue DNA (Matsui et al., 2016). The primer sequences used are as follows (5′ to 3′) (Fw/Rv; amplified gene):

16SDNA bacterial gene CATTCATGTTTCGAATCATTTCAAA/GAAACACGGACACCCAAAGTA; actin CGTCATCCATGGCGAACTG/GCTTCTTTGCAGCTCCTTCGT; CCL-2 TGCCCTAAGGTCTTCAGCAC/AAGGCATCACAGTCCGAGTC; TGF-β1 TGCGCTTGCAGAGATTAAAA/AGGTAACGCCAGGAATTGTTGCTA; IL-13 GACCAGACTCCCCTGTGCAA/TGGGTCCTGTAGATGGCATTG; IFN-γ CTTGGATATCTGGAGGAACTGGC/GCGCTGGACCTGTGGGTTGTTGA; IL-4 CATCGGCATTTTGAACGAGGTCA/CTTATCGATGAATCCAGGCATCG; IL-10 CCAGTTTTACCTGGTAGAAGTGATG/TGTCTAGGTCCTGGAGTCCAGCAGACTCAA.

### Western blot

For western blot, tissue samples were collected on Buffer RIPA (NaCl 150 mmol/L, Triton x-100 1%, sodium deoxycholate 1%, SDS 0.1%, EDTA 1 mmol/L, PMSF 1 mmol/L, leupeptin 1 μg/ml en Tris-HCl 50 mmol/L pH 7.4) and homogenized using Bio-Gen PRO200 homogenizer three times for 5 s at maximal speed. Protein samples were separated by SDS-PAGE on 10% polyacrylamide gels and electrotransferred to PVDF membranes using Trans-Blot SD (Bio-Rad) according to the manufacturer's instructions. Membranes were incubated with mouse monoclonal anti-β-actin (GenScript), or the already described anti-α-smooth muscle actin protein. Membranes were washed again and incubated with anti-mouse-HRP (Santa Cruz Biotechnology) or anti-rat biotinylated followed by streptavidin HRP. Bands were detected by enhanced chemiluminescence (Amersham) and quantified using LabWorksTM 4.6 (Image Acquisition and Analysis Software) and normalized to β-actin expression.

### Fibroblast and macrophage cell cultures

The previously characterized human foreskin fibroblast cell line FPA (De Campos-Nebel et al., 2010) was obtained from Servicio de Cultivo de Tejidos, Depto Virologia, INEI-ANLIS Dr. CG Malbran (Buenos Aires, Argentina) and was grown in Minimum Essential Medium supplemented with 10% FBS and 2 mM L-glutamine, pH 7.4. CD14 monocytes were isolated from peripheral blood mononuclear cells (PBMCs) using CD14 positive selection kit (Stem Cells Technology) and human monocyte-derived macrophages (HMDM) were obtained after 7 days of culture in the presence of rM-CSF as previously reported (Kusne et al., 2014).

The fibroblasts (1 × 10^5^) were seeded in 24-well flat-bottom-plates with or without poly-L-lysine treated coverslips, and placed in a humidified incubator at 37°C with CO_2_ (5%) for immunofluorescence or flow cytometry analysis. Fibroblasts were incubated with RPMI medium with 2% of FBS, LIC conditioned-medium, or LIC (MOI 10) for 48 h. Co-culture of FPA and HMDM assays were performed also in presence or absence of N-Acetyl-D-lactosamine (Carbosynth) as a Galectin-3 inhibitor (Gal-3i) (Jaquenod De Giusti et al., 2015).

### Immunofluorescence

Cells were fixed with 1% paraformaldehyde and permeabilised with 0.1% Triton X-100. After blocking with 6% BSA, cells were incubated for 1 h with a primary antibody against α-smooth muscle actin protein (Dako) followed by incubation with a FITC-conjugated secondary antibody (Life Technologies, Grand Island, NY, USA). Nucleus were stained with propidium iodide (Sigma-Aldrich, USA). Slides were analyzed with a Nikon E200 fluorescent photomicroscope (Nikon E200, Tokyo, Japan).

### Flow cytometry

To evaluate α-smooth muscle actin protein expression levels, treated fibroblasts were detached using trypsin/EDTA, fixed with 1% paraformaldehyde, permeabilised with 0.1% Triton X-100, and stained with a mouse antibody against α-smooth muscle actin protein followed by FITC-conjugated secondary antibodies (Life Technologies, Grand Island, NY, USA) or a matched IgG isotype as a negative control (BD Biosciences, San Jose, CA, USA) for 30 min at room temperature. Flow cytometry analysis was performed using a FACSCalibur flow cytometer (BD Biosciences, Franklin Lakes, NJ, USA) using FCS Express V3 software (De Novo Software, Los Angeles, CA, USA).

### Statistical analysis

All results are expressed as the mean plus SEM. A Student's paired *t-*test was used to determine significant differences between means, and *P*-values lower than 0.05 were considered statistically significant. When multiple groups were compared, a one-way analysis of variance (ANOVA) followed by the Tukey's multiple comparison test was used to determine significant differences between groups. Pearson correlation coefficient *r* was used for correlation analysis. All statistical analyses were performed using Prism 6 software (GraphPad).

## Results

### Macrophage depletion increased bacteremia and the subacute kidney bacterial burden but reduced subacute nephritis

**Figure 1:**
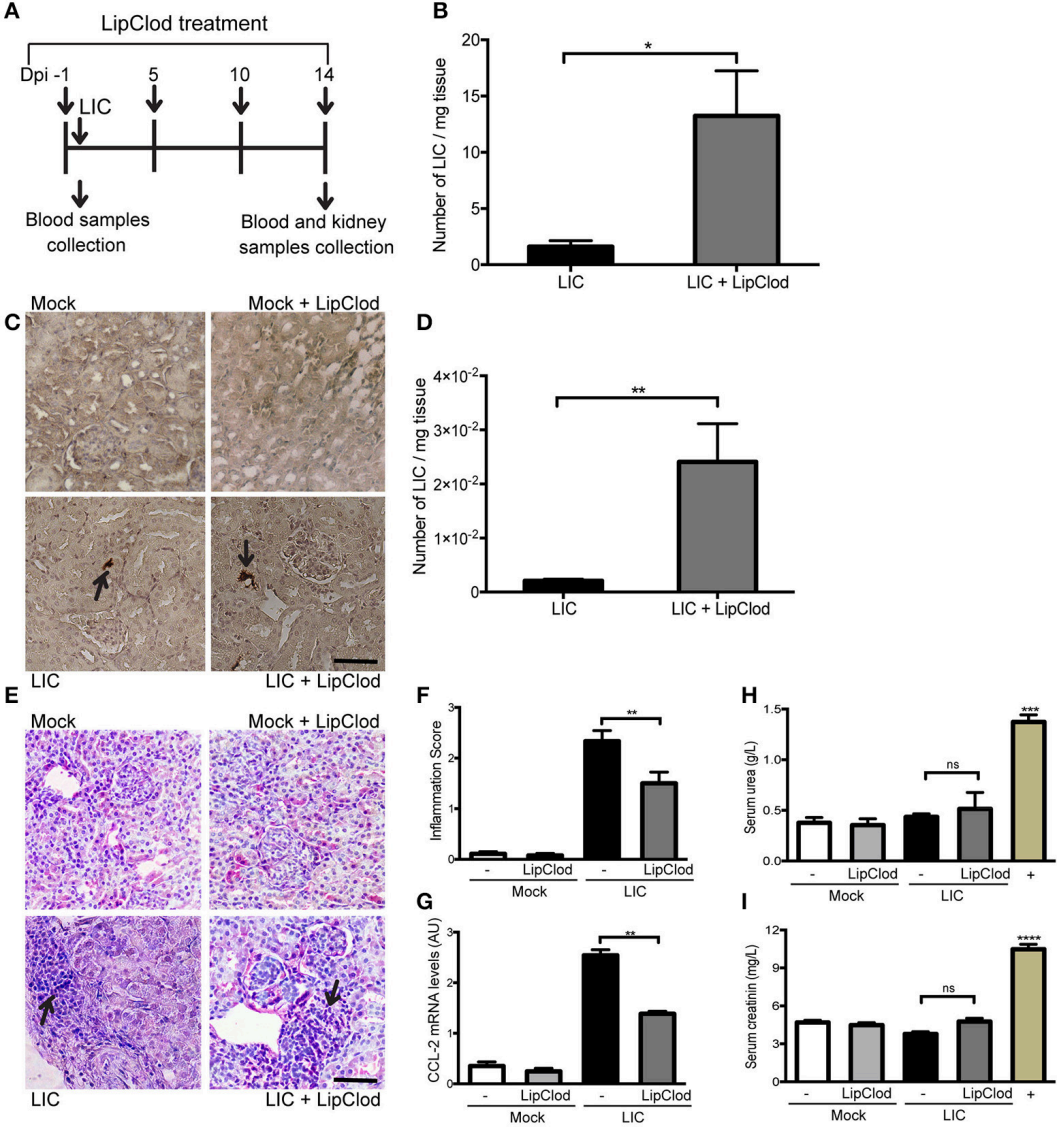
Depletion of macrophages resulted in a higher bacterial burden, reduced subacute nephritis, and unaltered renal function. **(A)** Treatment scheme for intraperitoneal depletion and infection in Mock, Mock + LipClod, LIC and LIC + LipClod groups of C57BL/6J mice. **(B)** Bacteremia levels at 1 day post-infection (dpi) were significantly increased in LIC-infected mice depleted of macrophages. **(C,D)** At 14 dpi, kidneys sections of all groups of mice were immunostained for LipL32 (the major *Leptospira interrogans* antigen) and also bacterial load in LIC + LipClod group resulted in higher levels compared to undepleted but LIC-infected mice. **(E–G)** Representative haematoxylin-eosin staining of kidney sections. An inflammation score was established by a pathologist and CCL-2 mRNA was quantified by qRT-PCR as a molecular marker of inflammation; β-actin was used as a housekeeping control. Significantly diminished levels of inflammation were detected in LIC + LipClod group. **(H,I)** Normal urea and creatinine levels in serum samples from all experimental groups were detected and assayed as a measure of renal functions at 14 dpi. Serum samples from LIC-infected gerbils were used as a positive control (+). In all cases, data represent assays of two independent experiments of groups of 6 animals, each. Bars indicate 50 microns; AU, arbitrary units; ^ns^*P* > 0.05; ^*^P < 0.05; ^**^P < 0.01; ^***^P < 0.001; ^****^P < 0.0001.

To clarify the role of macrophages in acute murine leptospirosis, we first studied the survival rate and bacterial burden in C57BL/6J mice with or without macrophage-depletion by LipClod treatment after *L. interrogans* serovar Copenhageni (LIC) infection (Figure 1A). All groups of mice had a 100% survival rate. Bacteremia at 1 day post infection (dpi) was significantly increased in LIC + LipClod mice relative to the LIC group (Figure 1B, *P* < 0.05). At 14 dpi, LipL32 immunostaining was detected in the kidneys (Figure 1C) and a higher bacterial load in LIC + LipClod mice which was confirmed by measuring bacterial DNA levels (Figure 1D, *P* < 0.01).

Analysis of kidney histopathology showed no histological abnormalities at 14 dpi in uninfected mice meanwhile LIC-infected mice exhibited a moderate interstitial nephritis that was significantly reduced in LIC + LipClod mice (Figure 1E), scoring 2.33 ± 0.36 and 1.5 ± 0.30, respectively (Figure 1F, *P* < 0.01). The pathological score was further supported by CCL-2 mRNA levels, used as an inflammatory marker, since expression levels were significantly lower in LIC + LipClod treated animals compared to LIC infected animals (Figure 1G, *P* < 0.05).

To determine whether macrophage depletion modulated renal function, the levels of urea and creatinine in the serum were measured at 14 dpi. Results showed that while there was a significant increase in infected gerbils (used as positive controls), no significant differences were found among mice groups (Figures 1H,I). These results suggested that kidney inflammation in our model was below the threshold necessary to elicit physiological kidney alterations such as those observed in gerbils, where kidney inflammation was clearly higher (data not shown).

Taken together, these results suggest that infection with LIC under macrophage depletion conditions results in an increased acute and subacute bacterial burden without impairment of renal function by the infection.

### Persistent depletion of macrophages resulted in increased chronic kidney bacterial burden and increased fibrosis

**Figure 2:**
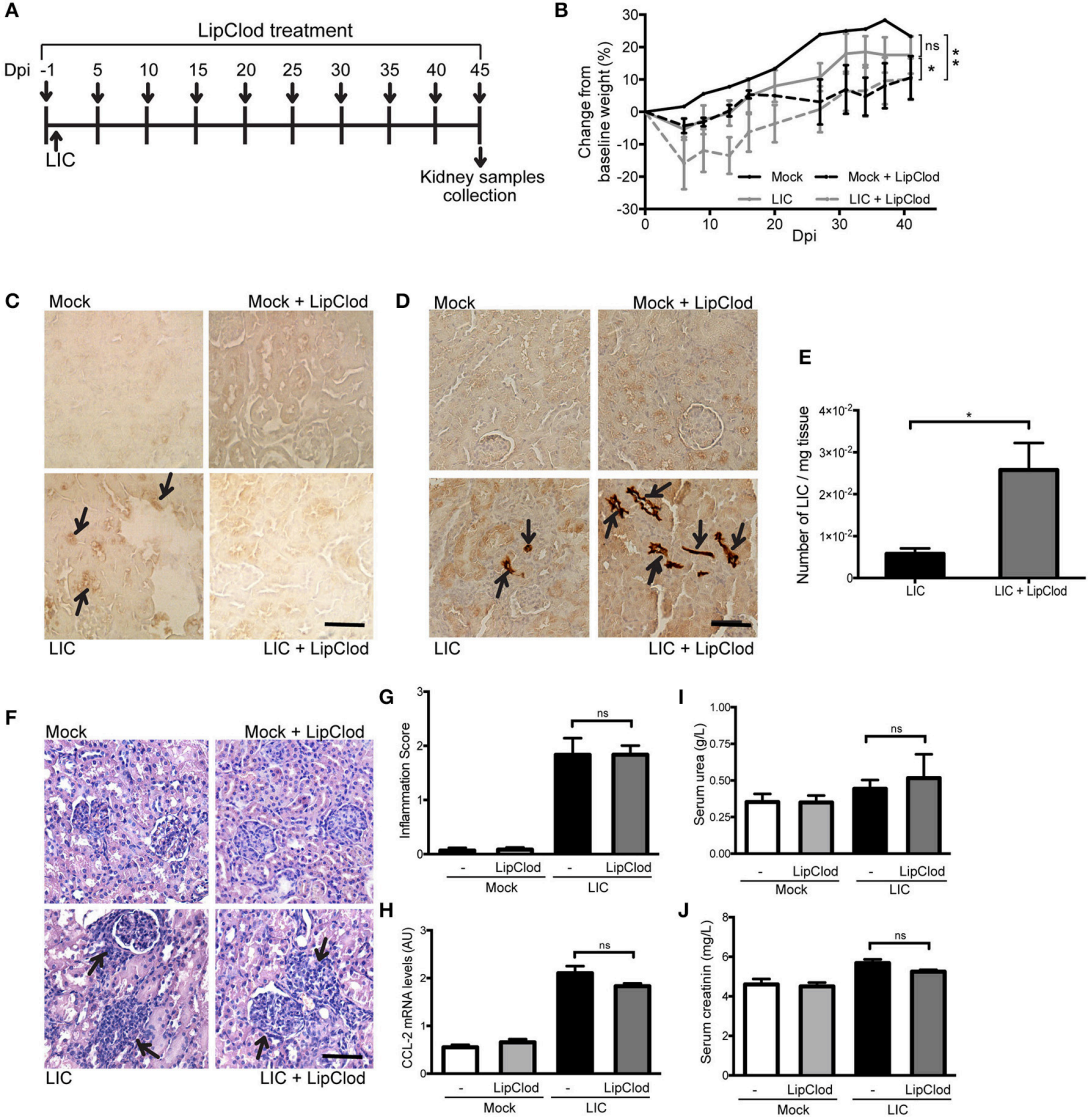
Depletion of macrophages resulted in an increased chronic renal bacterial burden and similar chronic interstitial nephritis. **(A)** Treatment scheme for intraperitoneal depletion and infection in Mock, Mock + LipClod, LIC and LIC + LipClod groups of C57BL/6J mice. **(B)** Percentage of body weight change in all groups of mice during the 45 days post-infection (dpi) showing that the LIC + LipClod group had significantly reduced weight gain. Immunostaining with an antibody against F4/80+ cells (macrophages) **(C)** confirming macrophage depletion or with anti-LipL32 (the major *Leptospira interrogans* antigen) **(D)** in kidney sections of all experimental groups of mice at 45 dpi. Depletion of macrophages in LIC-infected mice resulted in more presence of leptospiral antigen. **(E)** Higher bacterial load in kidneys of LIC + LipClod group was detected compared to undepleted LIC group. β-actin was used as a housekeeping control. **(F–H)** Representative haematoxylin-eosin staining of kidney sections. An inflammation score was established by a pathologist and CCL-2 mRNA was quantified by qRT-PCR as a molecular marker of inflammation; β-actin was used as a housekeeping control. Similar chronic inflammation was observed among experimental groups. **(I,J)** Normal urea and creatinine levels in serum samples from all experimental groups were detected and assayed as a measure of renal functions at 45 dpi. In all cases, data represent assays of two independent experiments of groups of 6 animals, each. Bars indicate 50 microns; AU, arbitrary units; ^ns^*P* > 0.05; ^*^P < 0.05; ^**^P < 0.01.

To determine how macrophage depletion affects the progression of this disease, the same experimental design used to study subacute leptospirosis was carried out for 45 days to study chronicity (Figure 2A). Body weight was used as a clinical parameter and showed a progressive increase over time in Mock uninfected control. By contrast, the LIC-infected animal groups exhibited a slight decrease in body weight over the first 10 dpi. However, these mice then started to gain weight and ended with close to the same weight as the control mice. The LIC + LipClod group, on the other hand, had significantly reduced weight gain, gaining only 10% of their original weight by the end of the experiment ending with similar values as the Mock + LipClod group (Figure 2B, *P* < 0.05 vs. LIC-infected mice, *P* < 0.01 vs. mock control). At 45 dpi, no macrophage infiltration was observed in the controls, but a moderate infiltration was associated with the nephritis in the LIC group that, as expected, was absent in LIC + LipClod mice (Figure 2C).

Immunostaining of LipL32 was significantly higher in the kidneys of LIC + LipClod mice (Figure 2D) and at least 10 times higher than at 14 dpi. This trend was confirmed by quantifying the bacterial load by q-PCR analysis (Figure 2E, *P* < 0.05, vs. LIC-infected mice).

Histopathology (Figure 2F) showed an absence of abnormalities in uninfected mice and a similar moderate interstitial nephritis in both infected groups, scoring 1.83 ± 0.50 and 1.84 ± 0.26 for LIC and LIC + LipClod mice, respectively (Figure 2G). This result was supported by the mRNA levels of CCL-2 (Figure 2H). Unexpectedly, no significant differences were observed in the parameters of renal function assessed (Figures 2I,J).

**Figure 3:**
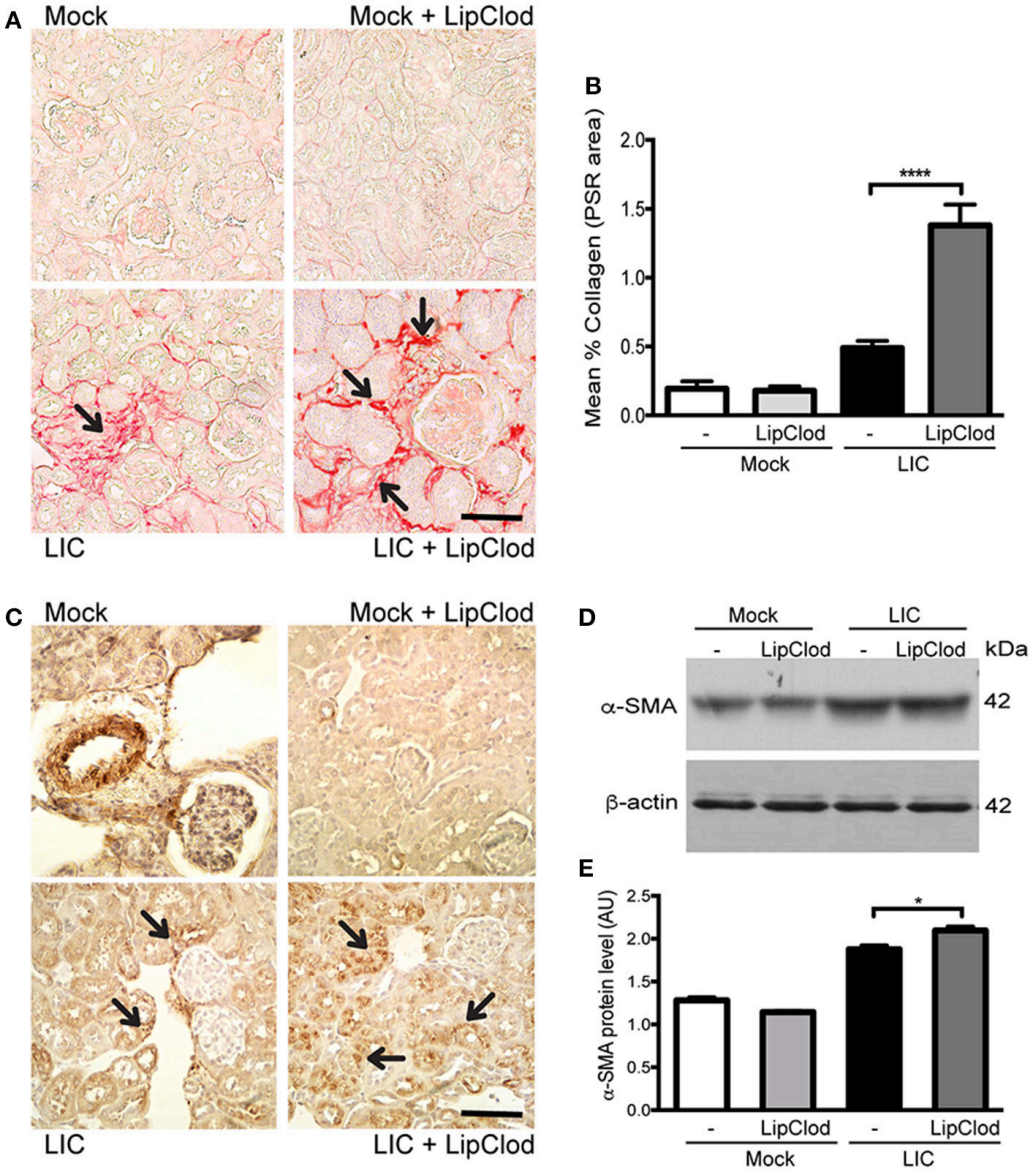
Depletion of macrophages resulted in an increased fibrosis. **(A,B)** Picrosirius red was used for collagen staining of kidney sections for all groups of mice at 45 dpi and digital quantification of fibrosis revealed significantly increased collagen deposition in the LIC + LipClod group of mice at 45 dpi. **(C–E)** α-smooth muscle actin protein immunostaining in all groups of mice and representative western blot image of α-smooth muscle actin protein in kidney samples from LIC and LIC + LipClod groups of mice at 45 dpi; β-actin was used as a housekeeping control. Data represent assays of two independent experiments of groups of 6 animals, each. For digital quantification, the samples derive from the same experiment and the gels/blots were processed in parallel. AU, arbitrary units; ^*^P < 0.05; ^****^P < 0.0001.

To determine whether macrophage depletion modulated chronic fibrosis, kidneys samples were stained for collagen with Picrosirius red (Figure 3A) at 45 dpi. None in the control group exhibited collagen deposits. By contrast, LIC + LipClod-infected mice had several foci of collagen deposits in the kidneys that were significantly higher in number and larger in size than those in the LIC group. Digital analysis of collagen area confirmed these observations with scores of 0.48 ± 0.10 and 1.38 ± 0.25 for LIC and LIC + LipClod, respectively (Figure 3B, *P* < 0.0001, comparing LIC to LIC + LipClod). Besides, in the LIC + LipClod group a positive correlation between Picrosirius red staining and bacterial burden was observed (*r* = 0.8982, *P* < 0.05). Enhanced α-smooth muscle actin protein (α-SMA) expression, as a parameter of myofibroblast activation, was observed in both groups of infected mice and enhanced in the LIC + LipClod group (Figure 3C). Western blot analysis confirmed the immunohistochemistry staining (Figures 3D,E, *P* < 0.05). Taken together, these results suggested that macrophages did affect the clinical course and that control of the bacterial burden was more important than inflammation to trigger chronic kidney fibrosis.

### Gal-3 disruption significantly increased the subacute bacterial burden and kidney inflammation

**Figure 4:**
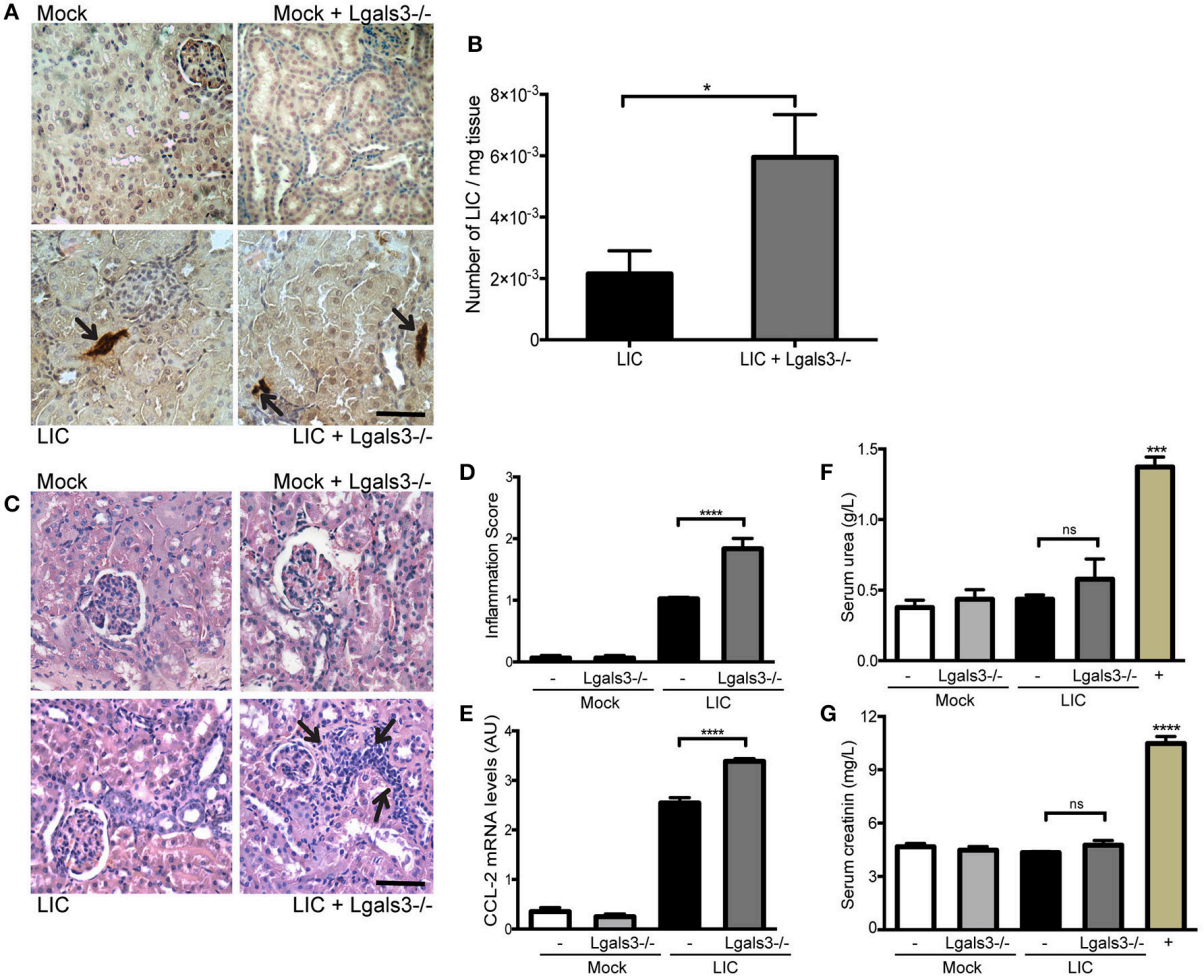
Genetic disruption of Gal-3 significantly increased the acute bacterial burden and acute kidney inflammation. **(A)** Immunostaining with an antibody against LipL32 (the major *Leptospira interrogans* antigen) in kidney sections of Mock, Mock + *Lgals3*^−/−^, LIC and LIC + *Lgals3*^−/−^ groups of mice at 14 days post-infection (dpi). **(B)** Absence of galectin-3 in LIC-infected mice resulted in higher bacterial load in kidneys compared to wild-type LIC group. β-actin was used as a housekeeping control. **(C–E)** Representative haematoxylin-eosin staining of kidney sections. An inflammation score was established by a pathologist and CCL-2 mRNA was quantified by qRT-PCR as a molecular marker of inflammation; β-actin was used as a housekeeping control. Acute kidney inflammation as well as significantly higher CCL-2 levels were detected in LIC + *Lgals3*^−/−^ group. **(F,G)** Normal urea and creatinine levels in serum samples from all experimental groups were detected and assayed as a measure of renal functions at 14 dpi. Serum samples from LIC-infected gerbils were used as a positive control (+). In all cases, data represent assays of two independent experiments of groups of 6 animals, each. Bars indicate 50 microns; AU, arbitrary units; ^ns^*P* > 0.05; ^*^P < 0.05; ^***^P < 0.001; ^****^P < 0.0001.

In order to study the role of Gal-3 in experimental murine leptospirosis, C57BL/6J *Lgals3*^−/−^ and their control littermate mice were used in a comparative study. There was an absence of spontaneous mortality in all groups. At 14 dpi, immunohistochemistry staining of LipL32 had increased in the kidneys of LIC + *Lgals3*^−/−^ mice (Figure 4A), which was confirmed by quantification of the bacterial burden by q-PCR analysis (Figure 4B, *P* < 0.05, vs. LIC-infected mice).

The histopathological analysis showed an increased, mild interstitial nephritis in LIC + *Lgals3*^−/−^ mice compared to LIC-infected mice (Figure 4C), with scores of 1.02 ± 0.16 and 1.83 ± 0.31, respectively (Figure 4D, *P* < 0.0001). These results were corroborated by the mRNA levels of CCL-2 (Figure 4E, *P* < 0.0001, LIC vs. LIC + *Lgals3*^−/−^ mice). Non-altered renal function was observed in any group (Figures 4F,G).

These results indicate that Gal-3 plays a role in limiting the bacterial burden and tissue inflammation during the acute phase of LIC-induced nephritis. Taken together, mice knockout for Gal-3 showed similar results to macrophage depletion.

### Gal-3 disruption increased the chronic bacterial burden and fibrosis, but do not affect inflammation

**Figure 5:**
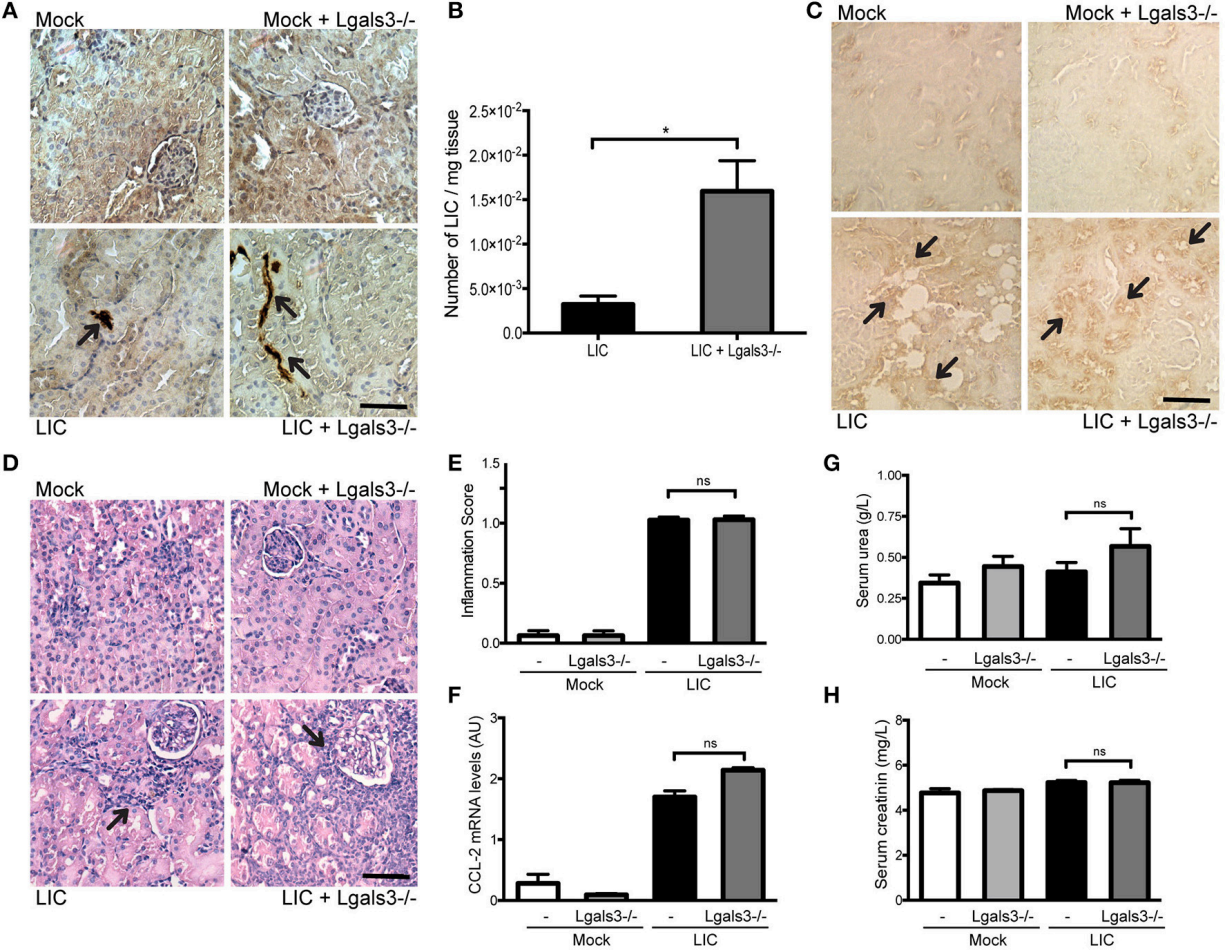
Genetic disruption of Gal-3 significantly increased the chronic bacterial burden. **(A)** Immunostaining with an antibody against LipL32 (the major *Leptospira interrogans* antigen) in kidney sections of Mock, Mock + *Lgals3*^−/−^, LIC and LIC + *Lgals3*^−/−^ groups of mice at 45 days post-infection (dpi). **(B)** Absence of galectin-3 in LIC-infected mice resulted in higher bacterial load in kidneys compared to wild-type LIC group. β-actin was used as a housekeeping control. **(C)** Immunostaining with an antibody against F4/80+ cells (macrophages) revealed its presence in both infected groups independent of galectin-3 absence. (**D–F)** Representative haematoxylin-eosin staining of kidney sections. An inflammation score was established by a pathologist and CCL-2 mRNA was quantified by qRT-PCR as a molecular marker of inflammation; β-actin was used as a housekeeping control. Similar chronic kidney inflammation was observed among experimental groups. **(G,H)** Normal urea and creatinine levels in serum samples from all experimental groups were detected and assayed as a measure of renal functions at 45 dpi. In all cases, data represent assays of two independent experiments of groups of 6 animals, each. Bars indicate 50 microns; AU, arbitrary units; ^ns^*P* > 0.05; ^*^P < 0.05.

At 45 dpi, immunostaining of LipL32 (Figure 5A) and quantification of the bacterial burden by q-PCR analysis (Figure 5B) showed that the bacterial burden was lower for the LIC group than the LIC + LipClod mice, but it was significantly increased in the kidneys of LIC + *Lgals3*^−/−^ mice relative to the LIC group (*P* < 0.05, vs. LIC-infected WT mice).

At chronicity, LIC infection induced similar slight macrophage infiltration in both wild type and *Lgals3*^−/−^ mice (Figure 5C). The histopathological analysis (Figure 5D) showed no abnormalities in uninfected mice and a similar discrete, interstitial nephritis in both of the infected groups, scoring 1.025 ± 0.10 and 1.03 ± 0.20, respectively (Figure 5E). This result was supported by the mRNA levels of CCL-2, which was used as an inflammatory marker (Figure 5F). Together, both of these values were similar but lower than those observed after macrophage depletion. As observed for subacute infection, at 45 dpi non-significant differences in renal function were detected between LIC and LIC + *Lgals3*^−^^/−^ mice (Figures 5G,H).

**Figure 6:**
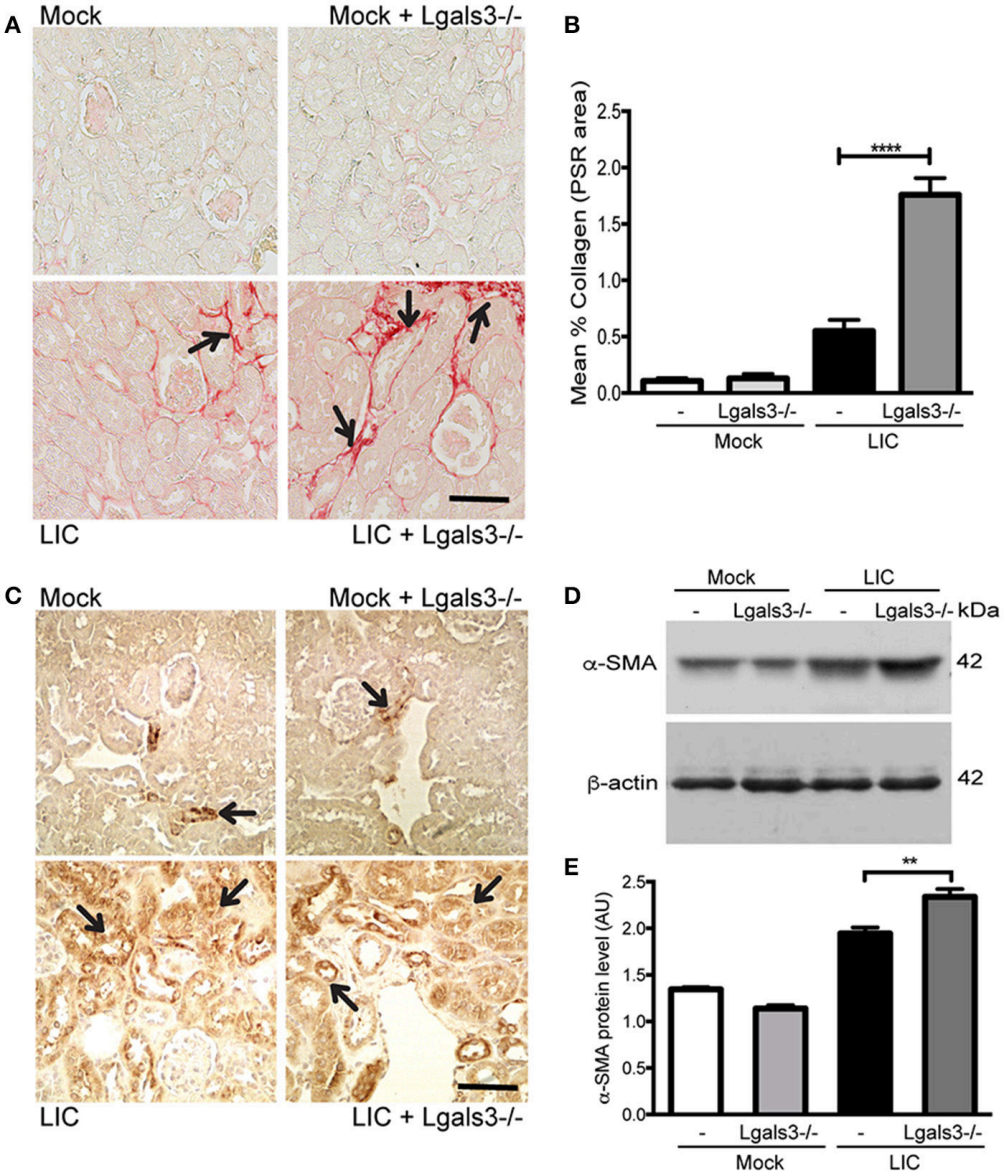
Genetic disruption of Gal-3 significantly increased the chronic fibrosis with similar chronic inflammation. **(A,B)** Picrosirius red was used for collagen staining of kidney sections for all groups of mice at 45 dpi and digital quantification of fibrosis revealed significantly increased collagen deposition in the LIC + *Lgals3*^−/−^ group of mice at 45 dpi. **(C–E)** α-smooth muscle actin protein immunostaining in all groups of mice and representative western blot image of α-smooth muscle actin protein in kidney samples from LIC and LIC + *Lgals3*^−/−^ groups of mice at 45 dpi; β-actin was used as a housekeeping control. Data represent assays of two independent experiments of groups of 6 animals, each. For digital quantification, the samples derive from the same experiment and the gels/blots were processed in parallel. AU, arbitrary units; ^**^P < 0.01; ^****^P < 0.0001.

To determine whether Gal-3 disruption modulated chronic fibrosis, kidney samples were stained for collagen with Picrosirius red (Figure 6A) at 45 dpi. None in the control group exhibited significant collagen deposits. By contrast, LIC + *Lgals3*^−/−^-infected mice had several foci of collagen deposits in the kidneys that were higher in number and larger in size than those in the LIC-infected mice group. Digital analysis confirmed these observations with scores of 0.55 ± 0.17 and 1.75 ± 0.21 for LIC and LIC + *Lgals3*^−/−^ mice, respectively (Figure 6B, *P* < 0.0001). Enhanced α-SMA expression was observed in both groups of infected mice (Figure 6C). The western blot analysis showed higher levels in the LIC + *Lgals3*^−/−^ mice (Figures 6D,E, *P* < 0.01).

### Fibrosis was not associated with TGF-β1 or IL-13 mRNA levels in the kidney

**Figure 7:**
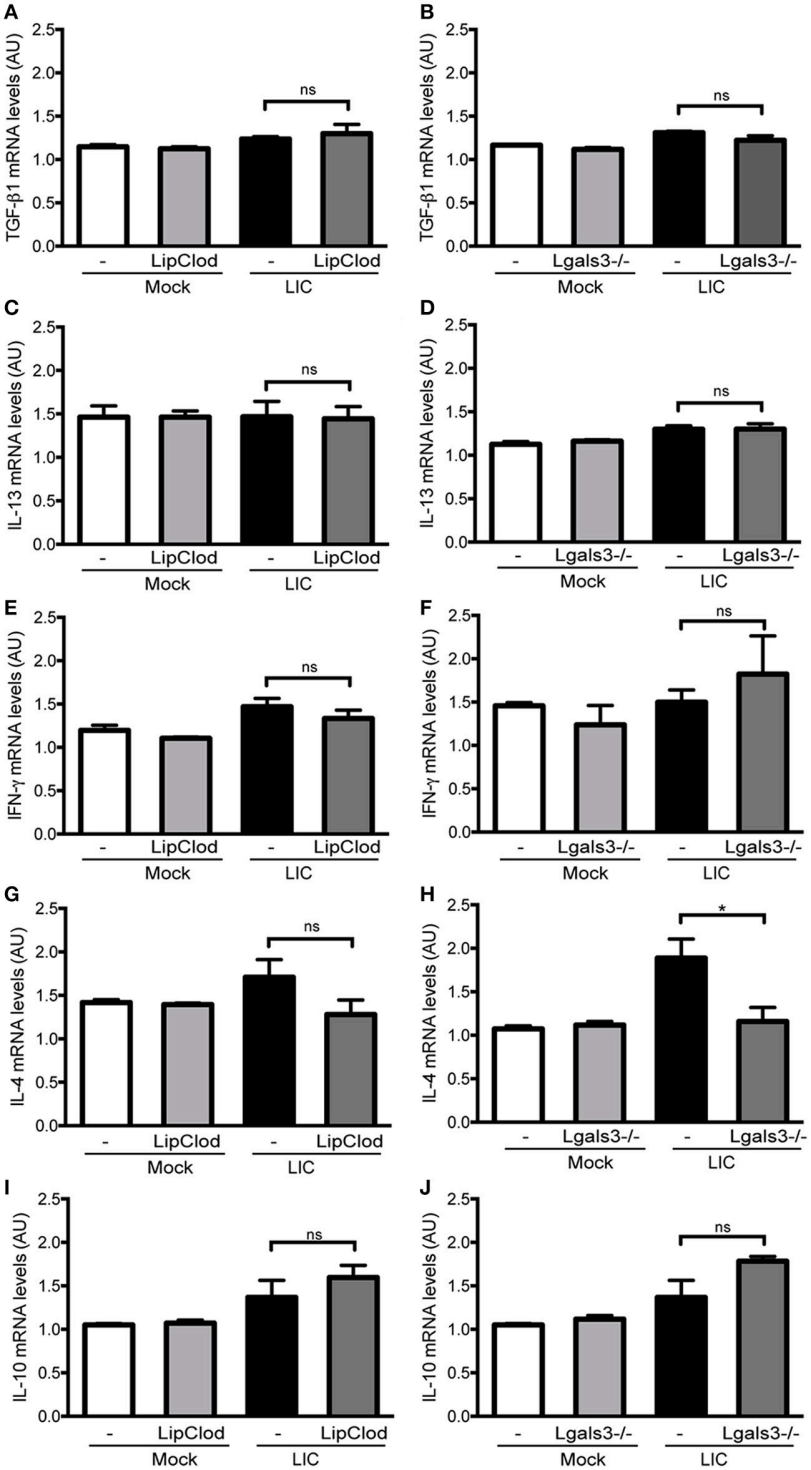
Cytokine mRNA levels in renal interstitial fibrosis triggered by LIC infection showing that fibrosis was not associated with TGF-β1 or IL-13 mRNA levels in the kidney. qRT-PCR analysis of TGF-β1 **(A,B)**, IL-13 **(C,D)**, IFN-γ **(E,F)**, IL-4 **(G,H)**, and IL-10 **(I,J)** mRNA levels in kidney samples from LIC, LIC + LipClod, and LIC + Lgals3^−/−^ groups of mice at 45 dpi. β-actin was used as a housekeeping control. Data represent assays of two independent experiments of groups of 6 animals, each; AU, arbitrary units; ^ns^*P* > 0.05; ^*^P < 0.05.

Previous studies have implicated some cytokines, including TGF-β1, as important mediators of renal fibrosis (Liu, 2011; Conway and Hughes, 2012). However, no significant differences were found in the TGF-β1 mRNA expression levels between LIC and the LIC + LipClod group or LIC and LIC + *Lgals3*^−/−^ mice at 45 dpi (Figures 7A,B). Similar results were found for the expression levels of IL-13, IFN-γ, and IL-10 (Figures 7C–F,I,J). In contrast, IL-4 mRNA expression was reduced in LIC + *Lgals3*^−/−^ mice while it remained unchanged in LIC + LipClod mice (Figures 7G,H).

Collectively, the analysis of mRNA expression levels for these cytokines revealed that they did not play a major role in triggering and/or developing LIC-induced fibrosis during the chronic phase of infection in the absence of macrophages or Gal-3.

### *Leptospira* triggered kidney fibrosis in experimentally infected and wild rats

**Figure 8:**
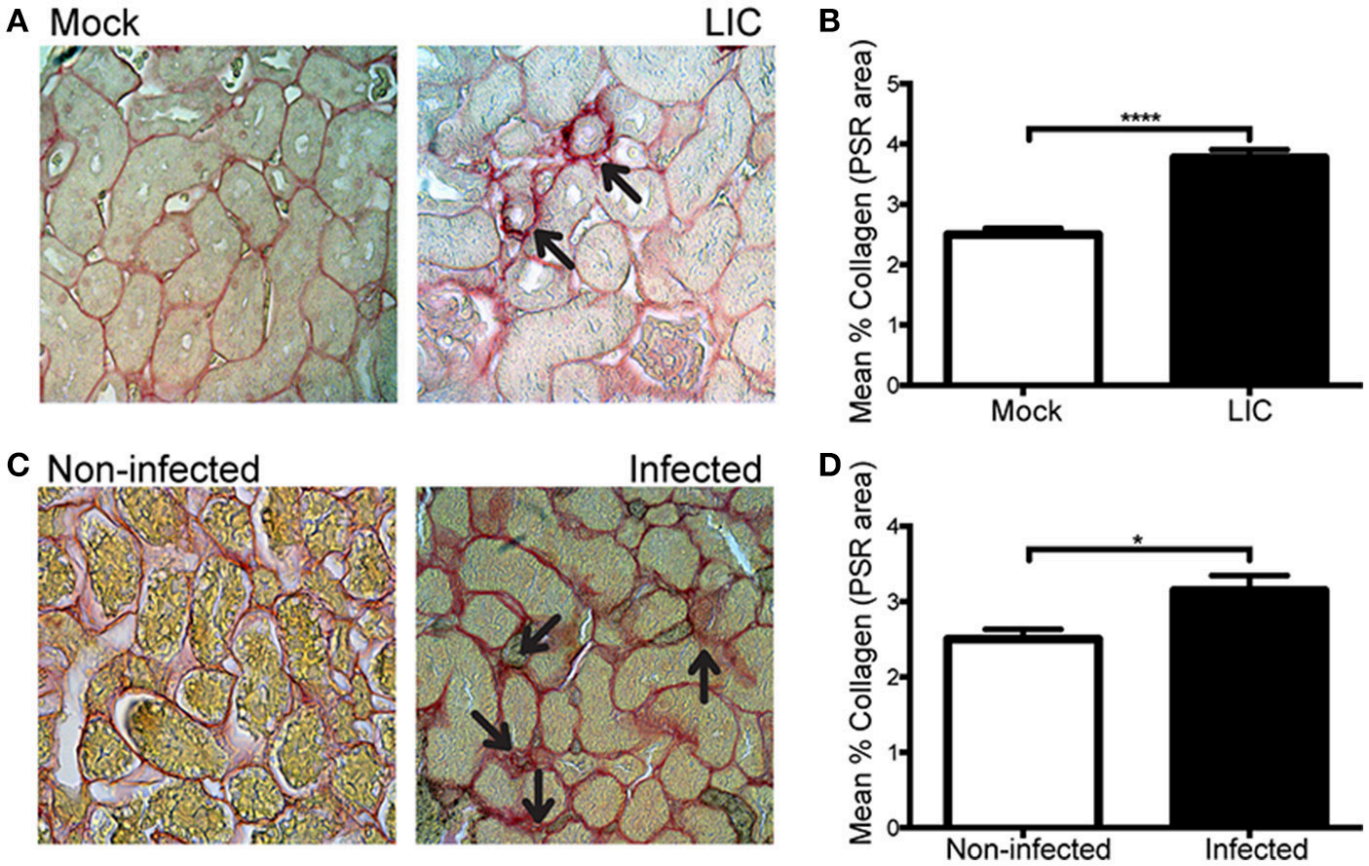
*Leptospira* triggered kidney fibrosis in experimentally infected and wild rats. **(A,B)** Picrosirius red was used for collagen staining of kidney sections for Mock, and experimentally LIC-infected rats and digital quantification of fibrosis revealed significantly increased collagen deposition in the LIC group at 45 dpi. **(C)** Wild rats were screened for *Leptospira* and 3 out of 48 animals were PCR-positive and Picrosirius red was used for collagen staining of kidney sections. **(D)** Digital quantification of fibrosis revealed significantly increased collagen deposition in the Infected (PCR-positive) compared with the Uninfected (PCR-negative) rats at 45 dpi. ^*^P < 0.05; ^****^P < 0.0001.

Next, in order to know if the enhanced collagen deposition observed in chronic murine leptospirosis was a species-related issue we extended our studies first to a rat experimental model, also used as a model of chronic leptospirosis. Picrosirius red staining of kidney samples from experimentally LIC-infected rats at 45 dpi showed more collagen staining than the mock-infected ones. Digital analysis confirmed these observations with scores of 2.49 ± 0.18 and 3.77 ± 0.15 for Mock and LIC rats, respectively (Figures 8A,B, *P* < 0.0001). Then, a similar study was performed in trapped-wild rats to confirm if fibrosis indeed happened in nature. A total of 48 animals were captured at the livestock farms, and all belonged to *Rattus norvegicus* species. Only 3 rats were positive for *Leptospira* according to PCR analysis. All of the 3 PCR-positive wild rats showed some degree of kidney inflammation and an enhanced renal collagen deposition when compared with PCR-negative rats (Figures 8C,D, *P* < 0.05).

### LIC activated human fibroblasts

**Figure 9:**
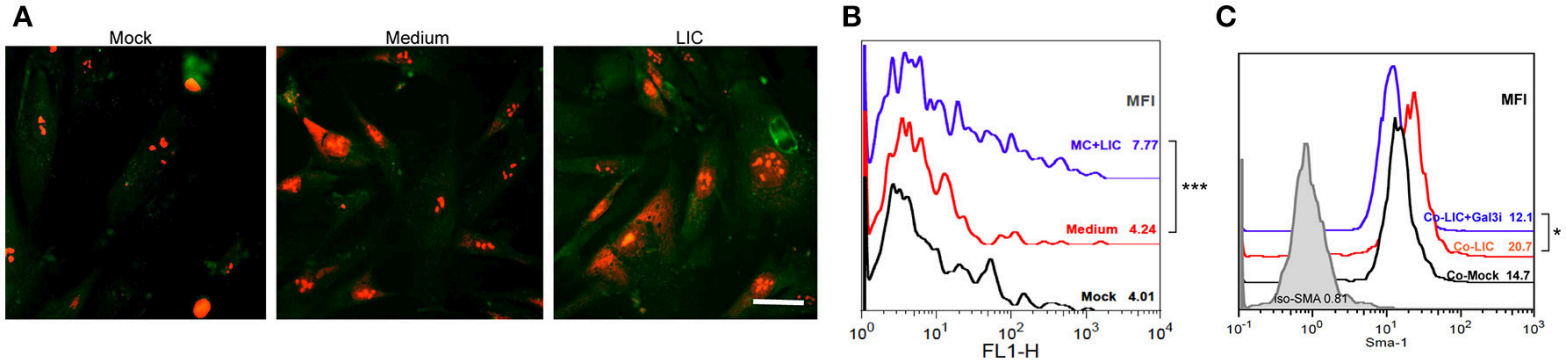
LIC activated human fibroblasts. **(A)** Immunofluorescent staining of α-smooth muscle actin protein (a specific marker of activated fibroblasts or myofibroblasts) in a primary cell line of cultured human fibroblast treated previously for 48 h with Mock, LIC conditioned-medium, and LIC. Propidium iodide was used for nuclear staining. **(B)** Representative FACS plots of SMA mean fluorescent intensity (MFI). **(C)** Representative FACS plots of SMA MFI of a co-culture (Co-) of fibroblast and macrophages infected or not for 48 h with LIC in absence or presence of a non-specific Galectin-3 inhibitor (Gal3i). Bar indicate 50 microns. ^*^P < 0.05; ^***^P < 0.001.

According to our results, chronic fibrosis did not seem to be enhanced by the presence of macrophages, Gal-3 expression or by a particular chronic cytokine. In contrast, we found that chronic fibrosis was associated with bacterial load. Therefore, we wanted to know whether LIC was able to induce fibroblast differentiation. Specifically, human fibroblasts were treated with LIC or LIC conditioned medium (negative control) and α-SMA protein was stained in cultured cells. The expression levels were quantified as a parameter of fibroblast conversion to myofibroblast, which is an accepted prerequisite of kidney fibrosis (Liu, 2011). Immunofluorescence (Figure 9A) and flow cytometry studies revealed an increased expression of α-SMA protein upon LIC treatment relative to control samples (Figure 9B, *P* < 0.001). In addition, we performed co-culture assays of human fibroblasts and macrophages LIC-infected in presence or absence of a Gal-3i. Results showed that LIC enhance the conversion to myofibroblast and that the Gal-3i blocked this effect (Figure 9C, *P* < 0.05).

## Discussion

In this study, we showed that macrophages and Gal-3 play a major role in controlling the LIC burden and its associated inflammation, but they are not involved in subsequent fibrosis. In agreement with previous studies, all C57BL/6J mice had a 100% survival rate (Fanton D'andon et al., 2014; Ferrer et al., 2014). The observation that the LipClod group of mice showed significant weight loss that was not associated with level changes in serum urea and creatinin suggests that macrophages are important mediators in the clinical evolution but not in kidney function of this disease.

Macrophages play an important role in controlling the bacterial burden and tissue inflammation during other spirochete infections. Their systemic depletion from mice using LipClod resulted in uncontrolled *Borrelia duttoni* growth and a 100-fold increase in the *Borrelia recurrentis* burden in blood, further supporting that these phagocytic cells play a critical role in controlling the spirochete burden (Larsson et al., 2009). Two pioneering studies were performed to clarify the role of macrophages in leptospirosis. Both studies used BALB/c mice and depleted macrophages by administering silica particles. One study found a similar outcome for the infection in non-depleted and depleted mice (Tu et al., 1982), while the second observed an increased sensitivity to *Leptospira* infection and an inhibition of bacterial clearance after depletion (Isogai et al., 1986). This work confirms and expands upon those results in a more susceptible mouse strain, as monocyte-macrophage phagocytic cells in C57BL/6J mice were found to play a critical role in the acute bacterial burden but subacute interstitial nephritis was reduced. Moreover, chronic depletion of macrophages resulted in an increased chronic kidney bacterial burden. Taken together, these findings strongly suggest that macrophages control the acute, subacute and chronic bacterial burden and have a key role in subsequent inflammation. This finding is particularly relevant as *Leptospira* sp. was reported to be able to replicate inside infected macrophages without any apparent cytopathic effects (Toma et al., 2011), leading to the possibility that infected macrophages participate in bacterial dissemination. Although our results do not discard this possibility, the observed increase in bacterial burden following macrophage depletion did not indicate that macrophages play a significant role in *Leptospira* sp. dissemination in mice.

Regarding the role of Gal-3 in experimental leptospirosis, our results showed that Gal-3 played a significant role in controlling both the subacute kidney bacterial burden and kidney inflammation. Moreover, Gal-3 also played a role in the chronic kidney bacterial burden. These data are in agreement with Gal-3 controlling initial *Leishmania* (Sato et al., 2014) as well as *Helicobacter pylori* infections (Subhash et al., 2016). Although several mechanisms have been implicated (including Gal-3 recognition of pathogen-associated molecular patterns such as lipopolysaccharide, microbe binding to target cells and chemoattraction by phagocytes Sato et al., 2014; Subhash et al., 2016), the specific mechanism(s) involved in leptospirosis require further studies to be clarified.

Our findings on kidney fibrosis were quite unexpected. It was not primarily related to the presence of macrophages as fibrosis was enhanced in their absence, even with a diminished interstitial nephritis. In a similar way, enhanced fibrosis was found in the absence of Gal-3 with a similar chronic inflammation, suggesting that the levels of Gal-3 were not critically relevant to kidney fibrosis, in contrast to that was shown in a non-infectious model of kidney fibrosis where macrophages and Gal-3 played a major role (Henderson et al., 2008). Recent studies showed that bone marrow-derived fibroblasts contribute significantly to the pathogenesis of renal fibrosis (Sun et al., 2016; Yan et al., 2016). Whether this mechanism is involved in leptospirosis should be determined in future studies. In addition, although TGF-β1 and IL-13 are typically involved in triggering the fibrotic processes, our present results showed that TGF-β1 mRNA levels were not up-regulated in *Leptospira*-induced chronic renal fibrosis. Surprisingly, neither IL-13 nor the other studied cytokines seemed to influence the LIC-induced fibrotic process. This does not necessarily mean that our model of fibrosis is TGF-β1 independent since TGF-β1 is regulated at many different levels and time points. Moreover, it has been shown that the addition of *Leptospira* to tubular epithelial cells induces collagen accumulation via a TGF-β1 dependent mechanism (Tian et al., 2006). Besides, Gal-3 deficiency did not affect macrophage infiltration and inflammation but was able to trigger kidney fibrosis that correlated with increased bacterial load, and together with the finding that non-specific Gal-3i reduce myofibroblast activation triggered by LIC, suggests that regardless of macrophage role, *Leptospira* sp. is responsible for the activation of α-SMA and might trigger kidney fibrosis directly either by forming biofilms in the lumen of the distal convoluted tubule and/or by the secretion of unknown molecule(s), perhaps other galectin/s, but requires further studies to confirm it.

A few recent studies have characterized renal fibrosis in experimental leptospirosis models. By using C57BL/6J and many genetically modified mice, d'Andon et al. showed that kidney fibrosis was iNOS-dependent but was independent of TGF-β1, TLR, and NLR signaling pathways. Furthermore, the authors indicated that murine kidney fibrosis was more highly correlated to the presence of *Leptospira* than with interstitial nephritis (Fanton D'andon et al., 2014). However, in that study, fibrosis was not abolished in the infected iNOS knockout mice suggesting that another unknown mechanism(s) exists to promote *Leptospira*-induced renal fibrosis. In contrast, a more recent study comparing chronic leptospirosis in hamsters to OF-1 mice found that fibrosis was only present in the chronically infected hamsters and was not correlated with TGF-β1 levels. It was related to the degree and persistence of inflammation rather than to the bacterial burden. The absence of murine fibrosis may be due to OF-1 mice being outbred, the use of different bacterial strains or inoculation of animals at a different ages (Matsui et al., 2016). In a previous study based on findings in Daf1^−/−^ mice that have an enhanced immune response and interstitial nephritis, we proposed that fibrosis did not correlate with TGF-β1 levels (Ferrer et al., 2014) and was more related to the degree of interstitial nephritis and the level of Gal-3 (Ferrer et al., 2014). However, the Daf1^−/−^ mice also had a significantly higher bacterial burden in their subacute kidneys so this result does not contradict our current findings.

Humans were considered unable to become persistently infected carriers of *Leptospira* spp. However, recent studies have challenged that paradigm, demonstrating asymptomatic renal colonization by either pathogenic or intermediate pathogenic *Leptospira* spp. (Ganoza et al., 2010). More interestingly, chronic kidney disease (CKD) was suggested to be a possible outcome of persistent *Leptospira* spp. infection in humans (Herath et al., 2014). In fact, a recent cohort study performed by Yang et al. was the first to demonstrate the association between CKD and *Leptospira* spp. infection (Yang et al., 2015). Our present study, which demonstrates that the bacterial burden and *Leptospira* itself can activate fibroblasts, highlights the importance of understanding the pathogenic mechanisms of leptospirosis and their link to kidney fibrosis.

## Data availability

The raw data supporting the conclusions of this manuscript will be made available by the authors, without undue reservation, to any qualified researcher.

## Author contributions

RG and MF designed the study. MF performed most of the experimental work. ES performed some Gal-3 studies. RG, MS, and MF wrote the main manuscript text and MF prepared Figures 1–9. AR performed the serum analysis. RD performed the pathology analysis. EC performed the FACS and the WB analysis. JN performed some chronic rats studies. AN and DM performed wild rats studies. All authors have reviewed the manuscript.

### Conflict of interest statement

The authors declare that the research was conducted in the absence of any commercial or financial relationships that could be construed as a potential conflict of interest.

## References

[B1] Allison A. C. Harington J. S. Birbeck M. (1966). An examination of the cytotoxic effects of silica on macrophages. J. Exp. Med. 124, 141–154. 10.1084/jem.124.2.141 4288309 PMC2180474

[B2] Bharti A. R. Nally J. E. Ricaldi J. N. Matthias M. A. Diaz M. M. Lovett M. A.. (2003). Leptospirosis: a zoonotic disease of global importance. Lancet Infect. Dis. 3, 757–771. 10.1016/S1473-3099(03)00830-2 14652202

[B3] Chen S. C. Kuo P. L. (2016). The role of galectin-3 in the kidneys. Int. J. Mol. Sci. 17:565. 10.3390/ijms17040565 27089335 PMC4849021

[B4] Conway B. Hughes J. (2012). Cellular orchestrators of renal fibrosis. QJM 105, 611–615. 10.1093/qjmed/hcr235 22139500

[B5] Costa F. Hagan J. E. Calcagno J. Kane M. Torgerson P. Martinez-Silveira M. S.. (2015). Global morbidity and mortality of leptospirosis: a systematic review. PLoS Negl. Trop. Dis. 9:e0003898. 10.1371/journal.pntd.0003898 26379143 PMC4574773

[B6] De Campos-Nebel M. Larripa I. González-Cid M. (2010). Topoisomerase II-mediated DNA damage is differently repaired during the cell cycle by non-homologous end joining and homologous recombination. PLoS ONE 5:e12541. 10.1371/journal.pone.0012541 20824055 PMC2932731

[B7] Faine S. (1988). Leptospirosis, in Laboratory Diagnosis of Infectious Diseases: Principles and Practice, eds Balows A. Hausler W. J. Ohashi M. Turano A. (New York, NY: Springer-Verlag), 344–352.

[B8] Fanton D'andon M. Quellard N. Fernandez B. Ratet G. Lacroix-Lamandé S. Vandewalle A.. (2014). *Leptospira interrogans* induces fibrosis in the mouse kidney through Inos-dependent, TLR- and NLR-independent signaling pathways. PLoS Negl. Trop. Dis. 8:e2664. 10.1371/journal.pntd.0002664 24498450 PMC3907306

[B9] Felzemburgh R. D. Ribeiro G. S. Costa F. Reis R. B. Hagan J. E. Melendez A. X.. (2014). Prospective study of leptospirosis transmission in an urban slum community: role of poor environment in repeated exposures to the Leptospira agent. PLoS Negl. Trop. Dis. 8:e2927. 10.1371/journal.pntd.0002927 24875389 PMC4038618

[B10] Ferrer M. F. Scharrig E. Alberdi L. Cedola M. Pretre G. Drut R.. (2014). Decay-accelerating factor 1 deficiency exacerbates leptospiral-induced murine chronic nephritis and renal fibrosis. PLoS ONE 9:e102860. 10.1371/journal.pone.0102860 25032961 PMC4102560

[B11] Ganoza C. A. Matthias M. A. Saito M. Cespedes M. Gotuzzo E. Vinetz J. M. (2010). Asymptomatic renal colonization of humans in the peruvian Amazon by Leptospira. PLoS Negl. Trop. Dis. 4:e612. 10.1371/journal.pntd.0000612 20186328 PMC2826405

[B12] Haake D. A. Levett P. N. (2014). Leptospirosis in Humans, in Leptospira and Leptospirosis, ed Adler B. (New York, NY: Springer), 65–98.

[B13] Hartskeerl R. A. Collares-Pereira M. Ellis W. A. (2011). Emergence, control and re-emerging leptospirosis: dynamics of infection in the changing world. Clin. Microbiol. Infect. 17, 494–501. 10.1111/j.1469-0691.2011.03474.x 21414083

[B14] Henderson N. C. Mackinnon A. C. Farnworth S. L. Kipari T. Haslett C. Iredale J. P.. (2008). Galectin-3 expression and secretion links macrophages to the promotion of renal fibrosis. Am. J. Pathol. 172, 288–298. 10.2353/ajpath.2008.070726 18202187 PMC2312353

[B15] Henderson N. C. Mackinnon A. C. Farnworth S. L. Poirier F. Russo F. P. Iredale J. P.. (2006). Galectin-3 regulates myofibroblast activation and hepatic fibrosis. Proc. Natl. Acad. Sci. U.S.A. 103, 5060–5065. 10.1073/pnas.0511167103 16549783 PMC1458794

[B16] Herath N. J. Kularatne S. A. Weerakoon K. G. Wazil A. Subasinghe N. Ratnatunga N. V. (2014). Long term outcome of acute kidney injury due to leptospirosis? A longitudinal study in Sri Lanka. BMC Res. Notes 7:398. 10.1186/1756-0500-7-398 24964804 PMC4080986

[B17] Isogai E. Kitagawa H. Isogai H. Kurebayashi Y. Ito N. (1986). Phagocytosis as a defense mechanism against infection with leptospiras. Zentralbl Bakteriol. Mikrobiol. Hyg. A 261, 65–74. 10.1016/S0176-6724(86)80063-3 3010604

[B18] Jaquenod De Giusti C. Ure A. E. Rivadeneyra L. Schattner M. Gomez R. M. (2015). Macrophages and galectin 3 play critical roles in CVB3-induced murine acute myocarditis and chronic fibrosis. J. Mol. Cell. Cardiol. 85, 58–70. 10.1016/j.yjmcc.2015.05.010 26002282

[B19] Ko A. I. Galvao Reis M. Ribeiro Dourado C. M. Johnson W. D. Jr. Riley L. W. (1999). Urban epidemic of severe leptospirosis in Brazil. Salvador leptospirosis study group. Lancet 354, 820–825. 10.1016/S0140-6736(99)80012-9 10485724

[B20] Kusne Y. Carrera-Silva E. A. Perry A. S. Rushing E. J. Mandell E. K. Dietrich J. D.. (2014). Targeting aPKC disables oncogenic signaling by both the EGFR and the proinflammatory cytokine TNFalpha in glioblastoma. Sci. Signal. 7:ra75. 10.1126/scisignal.2005196 25118327 PMC4486020

[B21] Larsson C. Lundqvist J. Van Rooijen N. Bergström S. (2009). A novel animal model of *Borrelia recurrentis* louse-borne relapsing fever borreliosis using immunodeficient mice. PLoS Negl. Trop. Dis. 3:e522. 10.1371/journal.pntd.0000522 19787030 PMC2742892

[B22] Lecour H. Miranda M. Magro C. Rocha A. Gonçalves V. (1989). Human leptospirosis–a review of 50 cases. Infection 17, 8–12. 10.1007/BF01643489 2921094

[B23] Liu F. T. Rabinovich G. A. (2010). Galectins: regulators of acute and chronic inflammation. Ann. N.Y. Acad. Sci. 1183, 158–182. 10.1111/j.1749-6632.2009.05131.x 20146714

[B24] Liu Y. (2011). Cellular and molecular mechanisms of renal fibrosis. Nat. Rev. Nephrol. 7, 684–696. 10.1038/nrneph.2011.149 22009250 PMC4520424

[B25] Matsui M. Roche L. Geroult S. Soupé-Gilbert M. E. Monchy D. Huerre M.. (2016). Cytokine and chemokine expression in kidneys during chronic leptospirosis in reservoir and susceptible animal models. PLoS ONE 11:e0156084. 10.1371/journal.pone.0156084 27219334 PMC4878748

[B26] Nally J. E. Wilson-Welder J. H. Hornsby R. L. Palmer M. V. Alt D. P. (2018). Inbred rats as a model to study persistent renal leptospirosis and associated cellular immune responsiveness. Front. Cell. Infect. Microbiol. 8:66. 10.3389/fcimb.2018.00066 29594063 PMC5861151

[B27] Pretre G. Lapponi M. J. Atzingen M. V. Schattner M. Nascimento A. L. Gómez R. M. (2013). Characterization of LIC11207, a novel leptospiral protein that is recognized by human convalescent sera and prevents apoptosis of polymorphonuclear leukocytes. Microb. Pathog. 56, 21–28. 10.1016/j.micpath.2012.10.002 23092690

[B28] Sarkar J. Chopra A. Katageri B. Raj H. Goel A. (2012). Leptospirosis: a re-emerging infection. Asian Pac. J. Trop. Med. 5, 500–502. 10.1016/S1995-7645(12)60086-8 22575986

[B29] Sato S. Bhaumik P. St-Pierre G. Pelletier I. (2014). Role of galectin-3 in the initial control of Leishmania infection. Crit. Rev. Immunol. 34, 147–175. 10.1615/CritRevImmunol.2014010154 24940913

[B30] Scharrig E. Carestia A. Ferrer M. F. Cédola M. Pretre G. Drut R.. (2015). Neutrophil extracellular traps are involved in the innate immune response to infection with Leptospira. PLoS Negl. Trop. Dis. 9:e0003927. 10.1371/journal.pntd.0003927 26161745 PMC4498591

[B31] Sikes R. S. Animal Care Use Committee of the American Society Of Mammalogists. (2016). 2016 Guidelines of the American Society of Mammalogists for the use of wild mammals in research and education. J. Mammal. 97, 663–688. 10.1093/jmammal/gyw078 29692469 PMC5909806

[B32] Subhash V. V. Ling S. S. Ho B. (2016). Extracellular galectin-3 counteracts adhesion and exhibits chemoattraction in Helicobacter pylori-infected gastric cancer cells. Microbiology 162, 1360–1366. 10.1099/mic.0.000322 27283429

[B33] Sun Y. B. Qu X. Caruana G. Li J. (2016). The origin of renal fibroblasts/myofibroblasts and the signals that trigger fibrosis. Differentiation 92, 102–107. 10.1016/j.diff.2016.05.008 27262400

[B34] Tian Y. C. Chen Y. C. Hung C. C. Chang C. T. Wu M. S. Phillips A. O.. (2006). Leptospiral outer membrane protein induces extracellular matrix accumulation through a TGF-beta1/Smad-dependent pathway. J. Am. Soc. Nephrol. 17, 2792–2798. 10.1681/ASN.2006020159 16928805

[B35] Toma C. Okura N. Takayama C. Suzuki T. (2011). Characteristic features of intracellular pathogenic Leptospira in infected murine macrophages. Cell Microbiol. 13, 1783–1792. 10.1111/j.1462-5822.2011.01660.x 21819516

[B36] Tu V. Adler B. Faine S. (1982). The role of macrophages in the protection of mice against leptospirosis: *in vitro* and *in vivo* studies. Pathology 14, 463–468. 10.3109/00313028209092128 6760092

[B37] Van Rooijen N. Hendrikx E. (2010). Liposomes for specific depletion of macrophages from organs and tissues. Methods Mol. Biol. 605, 189–203. 10.1007/978-1-60327-360-2_13 20072882

[B38] Van Rooijen N. Sanders A. (1997). Elimination, blocking, and activation of macrophages: three of a kind? J. Leukoc. Biol. 62, 702–709. 10.1002/jlb.62.6.702 9400810

[B39] Wunder E. A. Jr. Figueira C. P. Santos G. R. Lourdault K. Matthias M. A. Vinetz J. M.. (2016). Real-time PCR reveals rapid dissemination of *Leptospira interrogans* after intraperitoneal and conjunctival inoculation of hamsters. Infect. Immun. 84, 2105–2115. 10.1128/IAI.00094-16 27141082 PMC4936353

[B40] Yan J. Zhang Z. Jia L. Wang Y. (2016). Role of bone marrow-derived fibroblasts in renal fibrosis. Front. Physiol. 7:61. 10.3389/fphys.2016.00061 26941655 PMC4766307

[B41] Yang H. Y. Hung C. C. Liu S. H. Guo Y. G. Chen Y. C. Ko Y. C.. (2015). Overlooked risk for chronic kidney disease after leptospiral infection: a population-based survey and epidemiological cohort evidence. PLoS Negl. Trop. Dis. 9:e0004105. 10.1371/journal.pntd.0004105 26452161 PMC4599860

